# The Conundrum of Training and Capacity Building for People with Learning Disabilities Doing Research

**DOI:** 10.1111/jar.12213

**Published:** 2015-09-02

**Authors:** Melanie Nind, Rohhss Chapman, Jane Seale, Liz Tilley

**Affiliations:** ^1^ University of SouthamptonSouthamptonUK; ^2^ Carlisle People First Research TeamCarlisleUK; ^3^ University of ExeterExeterUK; ^4^ The Open UniversityMilton KeynesUK

**Keywords:** capacity building, inclusive research, learning disabilities, training

## Abstract

**Background:**

This study explores the training involved when people with learning disabilities take their place in the community as researchers. This was a theme in a recent UK seminar series where a network of researchers explored pushing the boundaries of participatory research.

**Method:**

Academics, researchers with learning disabilities, supporters and other inclusive researchers considered important themes arising from presentations about developments in participatory research. The paper emerges from critical reflection on these rich discussions.

**Results:**

A seminar series is a form of research training and capacity building, albeit a dynamic, interactive and collegial one. More formal training in research skills for people with learning disabilities is being developed but raises questions regarding the best contribution people with learning disabilities can make to the research process.

**Conclusion:**

There are various models of training for inclusive research, but these need to be reciprocal if they are not to undermine the inclusive goal.

## Introduction

Important developments are happening for people with learning disabilities involved in research. The 2014 special issue of JARID (volume 27 issue 1) showcased New Directions in Inclusive Research. It did so in a way that did not leave inclusive research unproblematized, instead asking questions of it, including questions about its importance and impact and about the boundaries between inclusive research and advocacy (Strnadová & Cumming 2014). This study arises from a similar questioning of participatory or inclusive research during a series of five seminars across two years in which people involved with doing and supporting research with (and for) people with learning disabilities addressed how the boundaries of this research were being pushed. The particular concerns were areas where there is still considerable scope for further development: the involvement of people with learning disabilities in data analysis (see Nind 2011) and the involvement of people with high support needs or profound impairment in research at all (see McLarty & Gibson 2000). The main findings from the seminar series are discussed elsewhere (Seale *et al*. forthcoming). In this study, we discuss a theme arising in the seminars that prompted particular concern in terms of the need for further attention: the conundrum of how the researched – people with learning disabilities – become the researchers with the necessary research skills and competences but without losing their unique perspective as people with learning disabilities – the object of the research.

The paper initially addresses how people ordinarily learn to become researchers. It moves to examine how learning to become a researcher might be different when starting from a position outside the academy and with marginalized social status as is the case for people with learning disabilities. The conundrum of training for people with learning disabilities is then explored and the importance of discussing it is argued. It is our contention that training for inclusive research is more complex than it may at first seem. We draw on literature in the fields of researcher education, participatory and inclusive research and on the contributions to the seminar series^1^ to tease out models of training and capacity and the challenges for making this fit with the aims of inclusive research.

## Building Capacity to Research – Learning to Become Researcher

Much of the discourse around capacity building in social science research assumes at least a fundamental grounding in research skills on which to build; to emphasize this, Trostle (1992, p. 1322) expresses a preference for referring to ‘“expanding research possibilities” rather than building research capacity’. He reminds us that capacity is subjective, context‐specific, mobile, and sometimes short‐lived, moreover that it concerns not just knowledge and skills about the technical and design aspects of research but also ‘knowing when the right question has been asked, and the right question has been answered’ (p. 1323). For Hammersley (2012) too, the capacity for research is a mix of knowledge, skills and virtues or dispositions. Accepting that training and capacity building (TCB) is the accepted term, Trostle's definition indicates that this refers to ‘a process of individual and institutional development which leads to higher levels of skills and greater ability to perform useful research’ (p. 1321).

Indeed, the TCB process is one of the expectations of a research council‐funded methodological, interdisciplinary seminar series like the one informing this paper. In TCB terms, the seminar series was intended not just to discuss the co‐production of research knowledge, but to co‐produce knowledge through its processes of discussion and reflection. This reflects the recognition that capacity is built in formal and informal learning spaces and in social contexts (Rees *et al*. 2007). Our aims were to critically examine the unacknowledged or underexplored tensions and challenges to what might be considered full or genuine participation by people with learning disabilities in research, and to stimulate innovative developments in methods by synthesizing achievements and acting as a catalyst for thinking and action.

Throughout the seminars, there was an unspoken understanding that everyone involved was learning through doing and through discussing. The research training of the academic researchers would have been a mixture of formal courses and apprenticeship‐style learning through the conduct of their supervised doctoral research, the move toward formal training rather than learning through researching coming relatively late in the UK. The people supporting research with people with learning disabilities could similarly testify from their learning journeys that ‘learning occurs in all social settings, not just those setting activities that are directed to particular kinds of intentions’ (Billett 2010, p.402). Learning to become inclusive researchers was often self‐taught, born of necessity (as discussed by inclusive researchers in Nind & Vinha 2012). Yet in the content of the presentations and discussions, considerable attention was being paid to the training needed to enable people with learning disabilities (or other marginalized individuals and groups) to cross the line from researched to researcher. This shifted attention away from that which is ‘learnt and not taught’ (Billett 2010, p;.403) to formal education and to learning in courses in the presence of a teacher with ‘the authority to determine that people designated as requiring knowledge effectively learn a curriculum taken from a pre‐established body of knowledge’ (Livingstone 2001, p. 2).

The lack of pedagogic culture around how research methods are taught and learned has been noted in relation to social science research generally (Wagner *et al*. 2011; Kilburn *et al*. 2014), thus creating the space in which trial‐and‐error in teaching research methods pervades (Earley 2014). This sits alongside the ‘messy and uncertain reality’ (Hammersley 2012, p. 3) of the research learning process. In the process of learning to do research, the value of experiential learning and learning by doing is well recognized (Kilburn *et al*. 2014). In the process of learning to do inclusive research, this is accentuated by the newness of the paradigm and the emphasis placed on the value of lived reality of, for example, people with learning disabilities in shaping the research goals and processes. It is through involvement in the various stages of research that people learn the ‘rudiments of research methods so they can assume collaborative roles in the research’ (Bagnoli & Clark 2010, p.103).

One aspect of inclusive research that marks it out as different from ordinary qualitative research is the efforts to make transparent what roles different contributors to the research have taken on. This is important for credibility (Walmsley 2004), and numerous papers therefore lay bare the inclusive research process (see e.g. Brookes *et al*. 2012; Butler *et al*. 2012; Chapman *et al*. 2014). This includes attending to the underlying training of the researchers. For instance, Strnadová *et al*. (2014, p. 14) explain one of factors interfering with equality of status and contribution for researchers with learning disabilities:

The majority of academic researchers gain their experiences and learn necessary research skills during their undergraduate and postgraduate studies, through mentoring from academic advisors, from professional development, by attending conferences and exposing their research to the critical review of their peers and by undertaking their own research projects. Therefore, in inclusive research initiatives, the academic researcher is at an advantage and cannot necessarily expect researchers with intellectual disability to already have research skills.

The solution for Strnadová *et al*. (2014) was to adopt an approach of providing training as and when needed, so that ‘training for specific skills occurred when it was determined that the skill was necessary to continue working on the project and where one or more team members had deficits in that particular skill.’ This is common in inclusive research with people with learning disabilities, but it can replicate as well as address inequalities in that the research team can be seen in terms of those with skills and those with deficits, those who train and those who are trained and so on. Despite the reference to deficits, Strnadová *et al*. (2014) worked to avoid reinforcing unequal positions in establishing a learning together approach of learning research skills being part of maturing as a team and preparing to conduct the study. Thus, ‘the entire research team conducted and participated in a research skills training programme’ (p. 15). Training was needed by academic researchers and by researchers with learning disabilities alike, because the training related specifically to the planned research project and to enhancing the research design. The training used a mix of discussion, role‐plays, reflection, and the use of technology (iPads) ‘to support the skills and competence of the four researchers with intellectual disabilities throughout the project’ (p. 15). Although the academics shared in doing the training, however, it was also designed by them as part of taking responsibility to evaluating and meeting the needs of the research team.

A similar emphasis on togetherness in training is seen in the recognition from Warren & Boxall (2009) of the lack of training for people wanting to do research in partnerships spanning social work academics and service users. Their *Researching Together* short course was intended to bring people together to learn from and with each other and to ‘raise questions about the “us” and “them” of learning, teaching and research as well as about the idea of “expert knowledge”’ (p. 287). The course, partly in response to government pushes for service user involvement in social work research, policy and practice, was deliberately ‘set up in such a way that the knowledges and experiences of service users were prioritised over the research literature and academic theory which underpins most social policy courses’ (p. 287). This challenged the ‘objectifying’ (p. 288) of service users that takes place when their knowledge is seen as an add‐on to research learning rather than embedded within it. This kind of learning together epitomizes the seminar series learning also, but it is quite different from the formal training in research skills, the imparting of academic knowledge, from one group to another that has been described and called for both in the literature and some of the seminars.

## The Literature on Research Training for Inclusive Research

Johnson (2009) sums up the training landscape in relation to inclusive research with people with learning disabilities, arguing that ‘while some organisations and individuals have provided research training to people with intellectual disabilities’, there remains ‘no coordinated approach to developing research skills, nor agreement about what this training should cover’. She asks, ‘What, if any, research training do people with intellectual disabilities want or need in order to be involved in inclusive research?’ linked to the goal to develop ‘a curriculum and resources which would support people with intellectual disabilities to undertake inclusive research’ (p. 252). While the literature on training for inclusive research is not extensive, this, including anecdotal accounts, can be interrogated. This evidence suggests that for many there is a lack of formal training in inclusive research, but that there are (at least) five models of TCB at work: the apprenticeship model, the lifelong learner model, the challenging inequality model, the addressing deficits model and the formal model. These are summarized in Table 1.

**Table 1:** Models of TCB

Model of TCB	Characteristics
Apprenticeship model	Novice working alongside more experienced researchers who model and mentor
Lifelong learner	Novice managing own need for ongoing training negotiating formal and informal opportunities to keep developing skills
Challenging inequality	Researchers with and without learning disabilities perceived as in need of TCB and learning together in equal footing
Addressing deficits	Novice seen as having skills or experience gaps and in need of training to address these
Formal	Novice taught by a teacher following a curriculum

In the *apprenticeship model*, the novice becomes a researcher by working alongside other more experienced researchers who act in the capacity of modeller of skills, mentor and critical friend. This is inferred as what is going on in the ‘participatory social process’ of collaborative data analysis described by Stevenson (2014, p. 31). Learning in an apprenticeship model can be regarded as a response to people's need for support rather than training as such, but it does build capacity.

In the *lifelong learner model*, researchers identify their own need for ongoing training. Taking responsibility for their own position in the market place, they register for courses as and when needed alongside seizing upon informal opportunities to continue to learn. This reflects the economic and personal dimensions of lifelong learning (Biesta 2006). Examples among researchers with learning disabilities might include the choosing of what aspects of the research process to engage in training about thereby exercising preference and learner control (Strnadová *et al*. 2014).

The *challenging inequality model* is characterized by the epistemological stance adopted by Warren & Boxall (2009) described above. It leads to researchers with and without learning disabilities learning together in recognition that both need support and training to conduct quality research (Walmsley 2004). Brookes *et al*. (2012), for example, describe their whole team examining together the connotations of the words used in their research about adult protection from abuse and looking together at some of the literature; they talk about this as learning lessons but with no one party taking on the role of teacher.

Penultimately, there also remains a strand of training that we call the *addressing deficits model*. This emerges from a refusal to sidestep the basic difference in preparation for research between academic researchers and collaborating researchers with learning disabilities; whether using the terminology of deficits or not, it attempts to address them. Johnson (2009), for instance, argues that people with intellectual disabilities need research training to take on new roles in conducting research and she describes experience of workshops incorporating role‐play and practice. Garcia Iriarte *et al*. (2014) refer to filling skills gaps and Strnadová *et al*. (2014, p. 20–21) argue that, when adopting aspects of traditional research, training for ‘colleagues with intellectual disabilities is necessary, so they are not disadvantaged in their skills and understanding, as the academic researchers have all had formal training on the research process’. There is a perceived relationship, therefore, between addressing skills deficits, and bringing researchers with and without learning disabilities onto a more equal footing. It is important that the deficit may be one of experience as well as training (Strnadová *et al*. 2014), social rather than inherent.

The formality and content of the training described in the literature varies greatly with some examples of our final model of *formal training*. This *formal training model* often but not always overlaps with the *addressing deficits model*, but characteristically always involves a teacher and a curriculum. In the Australian context, for example, Strnadová *et al*. (2014) outline a fifteen‐week training programme, entitled ‘Welcome to our class’ (p. 18) in which participants spent ninety minutes per week on content including problem formulation, the importance of reflection and self‐reflection, research planning and scheduling, ethics, recording, interviewing and communicating results. While researchers with and without learning disabilities were learning together, their learning outcomes were differentiated so that while the former learned about core elements of research, the latter learned about things like research support and technological tool use. In the Irish context, university researchers provided training through workshops providing ‘basic understanding of research methods’ but also building research teams, apparently being both proactive and responsive in their delivery model (Garcia Iriarte *et al*. 2014; p. 152). There is also often an interaction between different kinds of training coming together in mixed packages. For example, Bentley *et al*. (2011) recount having training workshops with Jan Walmsley at key points, but also doing their own practising and problem‐solving in their training‐as‐and‐when‐needed approach.

## Seminar Discussion about Training for Inclusive Research

Training in inclusive research was not the main focus of any of the seminars. The daylong events were dedicated (in this sequence) to the following: *Scoping the boundaries of participatory research, Participatory data analysis, Participatory research with people with high support needs, Exploring issues of transfer of knowledge about inclusive research*, and *New ideas and next steps*. Across these main organizing topics for the series, the theme of research training repeatedly emerged. This was not always direct and explicit, but reflection on the training implications was repeatedly prompted by each topic of conversation. In the initial scoping seminar, Gordan Grant^2^ posed the question of whether it is easier to talk about the processes of inclusive research than it is to talk about its products. This in turn says something about where the efforts lie in relation to TCB for inclusive research. Much more attention has been paid to the ethical/moral/political case for involving people with learning disabilities in research (with consideration given to how this happens) than to what this means for the quality of the research conducted in terms of rigor or theoretical robustness, for example (see Nind & Vinha 2012). On this theme, one participant identified the tension between understanding things (the academic agenda) and changing things (the self‐advocacy agenda). Again, this has implications for the nature of the TCB that is called for. Similarly, more is made of academics wanting to involve self‐advocates in their activity than the other way round. This has led to academics seeking to train self‐advocates in research skills perhaps more than self‐advocates seeking to train academics in advocacy/listening skills. Following her major role in the development of inclusive research, Jan Walmsley argued in the opening seminar that – for inclusive research, life experience is not enough – knowledge and skills are needed too.

Pushing the boundaries of participatory data analysis was sometimes seen to require particular development of new skills. Gudrun Stefánsdóttir, with students Ólafur Snævar Aðalsteinsson and Embla R. Hakadóttir, described a process of formalizing dialogic learning experiences in an example of diploma and degree students, with and without learning disabilities, learning together on courses at the University of Reykjavik. This involved them in shared research projects and with this in joint analysis, but most importantly these students were learning and using methods together in what we have referred to above as the *challenging inequality model*. In this case, the *formal model* and the *challenging inequality model* coalesced. Also somewhat formalized was the Irish Inclusive Research Network approach described by Marie Wolfe and collaborators in that the academics recounted running workshops to recruit and train people with learning disabilities to run focus groups, and then the researchers with learning disabilities learned through doing aspects of the analysis alongside and following the academics. The approach described by the Carlisle People First Research Team to learning data analysis in contrast was less formal and more immersive and oriented toward practical problem‐solving. Equally, Val Williams and Andrew Barbour talked about de‐mystifying data analysis, challenging the idea of it being a separate or precious stage of research that is hard to learn, instead valuing the use of direct experience to reflect on data.

It was in the realm of the third seminar on including people with high support needs related to profound or multiple impairments where people were least well equipped in terms of the training in research skills they had experienced. Katherine Runswick Cole described a model largely absent in the learning disability inclusive research literature (and therefore not discussed above) of researcher‐in‐residence. Here, the academic researcher becomes immersed in and responsive to the environment of the learning disability organization. Hence, they also become a highly contingent resource for TCB as needed. Mostly though, researchers working to include those with the most complex impairments were by necessity self‐taught (as Debby Watson described), supported in their learning journeys by advisory groups and the like helping with their process of reflection and development. They might have learned about creative methods (such as in the accounts of Hilra Vinha and Sue Ledger and collaborators), or enabling technologies (as in the account of Andy Minion and Ajay Choksi), but the training resource for these researchers was primarily the challenges posed by the research situation and their readiness to adopt a can‐do attitude and problem‐solving orientation. For Nicola Grove, story‐telling was understood as an advocacy process, a research process and a learning process in that telling one's story is a route to insight. Her portrayal of stories as co‐constructions with people with high support needs helpfully pushed the boundaries of what we might understand by research and by TCB.

Research training was explicitly discussed in the fourth seminar in which people shared their experiences of doing research with children and young people, with mental health service users, and with older people as well as people with learning disabilities. ‘Training away the barriers’ was a major theme in the presentation from Toby Brandon and Caroline Kemp about their National Institute of Health Research study involving mental health service users, carers and academics. They avoided the ‘co‐researcher’ terminology used in other seminars, arguing that ‘you are either a researcher or you're not’, and that being a researcher meant being trained as one. They described a formal ten‐week certificated training course put on for non‐academics in their project, including an option to continue for an extra 4 weeks and then the option to work as researcher on the project. The formality of this training was emphasized by students on it gaining university credits from the final assessment in the form of a reflective essay following on from the aim to build confidence, rapport, teamwork and skills. The presenters discussed the need for training to challenge the idea that research is what other people do and address the barriers to training involved with research being associated with the academic domain. The concern was not just about training as and when needed, but with quality in the course and among the tutors, the university becoming a community resource for people in supporting them in learning how to learn through techniques such as buddying, mentoring and creative writing. The emphasis in relation to course content was on quality and values in research, doing it responsibly and safely, and developing research skills in situ responsive to needs. Just as seminar participants had been affirming the positive impact on individuals of being involved in doing research, here there was an affirmation of the impact of training on individuals’ confidence and identity. This coalition of an *addressing deficits* and *formal model* of training though was recognized to be expensive and only possible with generous grant funding.

In contrast, Sally Holland, in describing supporting ‘young people with extraordinary lives’ to do research, talked about learning by doing. She described fun ways of trying out methods and role‐playing proposals but ultimately focused not on the learning needed by the young people, but on that needed by her and the other academics involved. She reflected on how they had to learn to slow down their rush to see the research done and also on their skill deficits, compared with the young people, in competent use of technology. For these researchers, learning together experiences needed to be informal, active, relevant and engaging (see Holland *et al*. 2010).

An alternative position was taken by Craig Hart, self‐advocate researcher talking about the study led by Central England People First of their history. While the talk here was of much learning along the way (formal and informal) about oral history, the difficult terrain was seen as being the decisions the group had to make along the way. There was no direct training available for this – the group had to work their way through the challenges as they faced them. This had echoes of both the *lifelong learner model*, and also the *apprenticeship model*, as the research group were open to learning from the experience of Jan Walmsley and also for entrusting her with using her academic skills for parts of the research where they were needed. This is an alternative to engaging in training to fill skills gaps but one that requires considerable trust in the academic working for the good of the self‐advocates and under their control (see also Walmsley & The Central England People First History Project Team 2014). A similar approach was taken by self‐advocate researcher John Dias and his non‐disabled co‐researcher who worked on an equal pay job‐share to conduct the war memories study, but with the added formal element of them both attending a university oral history course for community researchers gaining formal certification (Dias *et al*. 2012).

Reflection on the seminar series has resulted in our seeing an enriched the picture of models of TCB from that teased out from the literature. Thus, added to the apprenticeship, lifelong learner, challenging inequality, addressing deficits and formal models are the following models which may be overlapping:

1. An *inclusive immersion model* in which aspects of the inclusive research are learned through an immersion in the research environment and its particular challenges within the distinctive context of the extra accountability and political sensitivity of inclusive research. What is distinctive from the apprenticeship model is that here there is no expert for the novice to learn from, just problems to learn through.
2. A *dialogic model* as in the seminar series itself in which inclusive researchers are able to learn through engaging with and testing each other's knowledge contributions.

Also adding to the complex picture of TCB for inclusive research is the observation that examples of an *apprenticeship model* evident in the regular research literature were sparse in the seminar series presentations and discussion; this may reflect a scarcity of available contexts suited to this kind of learning, a lack of mentors or sensitivity about who the experts really are. Moreover, the kind of lifelong learning seen in practice was shaped by the lack of available formal training and a tendency for inclusive research teams not to start from the research literature as a source of learning, mostly because of the accessibility difficulties associated with academic literature for people with learning disabilities. Several inclusive research teams have their roots in the self‐advocacy movement, and as such, there are already ‘porous boundaries’ (Chapman 2006) between the roles adopted by self‐advocates and supporters. This may mean that skills are melded and temporally and contextually dynamic (Chapman 2006). The nature of inclusive research demands that those involved learn to be a particular kind of researcher; this is as much about dispositions, stances and decisions as it is about skills. There is a literature to support the learning of these, but finding ways to make this practically accessible and politically acceptable as a reference point for research in which lived experience is dominant is a challenge.

## The Conundrum – up‐Skilling While Avoiding Training for the ‘Mini‐me’

The point of inclusive research (or at least one of the key points) is to bring new voices to the fore and different perspectives into dialogue (Nind 2014). This involves valuing our different life experiences. However, often the manifestation of these ideas is that members of the group who are the focus of the research (for this paper, people with learning disabilities) become researchers in their own right. This is where the demand for research training comes from. As Johnson (2009, p. 255) concluded, ‘While the lived experience of people with intellectual disabilities is central to inclusive research, it is also important that they be able to access training and skills when they are needed or wanted’. With TCB (whatever the model), insiders occupy new roles looking in as researchers, with their insider standpoint combining – or battling – with a new standpoint and lens as researcher.

This is best explained using the example of two differently experienced academic researchers (Irene & Niki) and two differently experienced researchers with learning disabilities (Gary & Amanda) collaborating in a study of the cancer experiences of people with learning disabilities (Butler *et al*. 2012). Irene, the lead academic reflects:

Before we started, I thought the preparation Gary and Amanda needed was ‘training in how to run focus groups’. How to facilitate a group and how to keep our own opinions out of it. I had attended an intensive 2 day course on facilitating focus groups and wanted to share my newfound wisdom… but it soon became clear that Gary and Amanda's role was very different from mine, and very different from what the text books recommend. In order to facilitate effective sharing within the group, what was needed was not Gary and Amanda's impartiality. On the contrary: their facilitative power lay in their ability to share of themselves within the group, to give their opinion about participants’ contributions and to resonate with them. (p. 141)

This is one of the rare examples in which the disadvantages of training are acknowledged. In reference to another study, Bigby *et al*. (2014, p. 57) discuss the potential for skills training for researchers with learning disabilities to be redundant:

There was no formal research training that is often found in other reports of inclusive research. As one member said, when asked what skills training she had been given, “What skills? We are the history, we do not need skills, but we did not get skills, they are already there” (Mins, 210311). She is referring not so much to her knowledge about the history that a research informant might bring, but to her capacity to use this knowledge and her social skills as part of the group that conducted interviews. This involved questioning interviewees about their version of events or pressing them for more information. Similarly, the academics brought their networks and own lived experiences of disability policy in Victoria, as well as more formalized research and organisational skills.

In the seminar series, Val Williams and Andrew Barbour, from their influential roots in self‐advocacy, similarly saw the value of an untrained, alternative approach to interviewing. Interestingly, Butler *et al*. (2012) were able to see the value of taking an untrained, alternative approach, but only when the researchers with learning disabilities were not leading the focus groups.

It may be, then, that it is leading research, rather than shaping it that drives the desire for formalized training. But as is plain from the reflection of Butler *et al*. (2012), the status of trained researcher has an allure: Contrasted with training ‘on the job’ and ‘learning by trial and error’, they argue that ‘it would be wonderful to be able to offer formal research training and a qualification to people with learning disabilities interested in becoming researchers’ (p. 142). This raises the question of what agenda the training serves: Is it to make the researchers more equal, to fill skills deficits, or to provide socially valued roles and status?

The conundrum is that if, as inclusive researchers, we value differences, then we should not inadvertently train them away and thereby lose the very sense of differences in dialogue that we were seeking. There is a danger that if unchecked and unproblematized, a drive toward training people with learning disabilities as researchers could be counter‐productive. It could push an agenda in which academics are implicitly saying to people with learning disabilities that for this to work you need to be more like me – know what I know. We know from the seminar participants that research training increases confidence and skills and this is of value. It may also close the training/skills gap a little, but it also changes the dialogic space of inclusive research. It may help some people with learning disabilities to cross a divide – from researched to researcher – but it is important not to lose sight of the relationship with other people with learning disabilities who remain in some way the researched. This paper is not an argument against TCB, but it is a call for pause to look at the unintended consequences of formalizing TCB as driving a shared language and research perspective.

Pushes to formalize training in research can equate to pushes for learning more intensively – in a ten week or even a half‐day course. This goes against the myriad of messages in the literature and from seminar participants that inclusive research – and the learning that it involves – takes time. There are dangers too that formalizing the training sets tighter parameters around who can participate in it, inevitably excluding some of the people who benefit from slower, more creative engagement. We know of research projects that did not happen because ethics committees did not see researchers with learning disabilities as sufficiently skilled and robust. Just as inclusive research is concerned with justice and a desire not to select people out of research (see Griffin & Balandin 2004), selecting people out of research TCB through a narrow model of training is to be avoided. Similarly, just as there have been warnings against working exclusively with research ideas that are accessible (Ramcharan *et al*. 2004), our reflections on our own TCB in the seminar series lead us to warn against working exclusively with research methods in which people with learning disabilities can be ‘trained’. We acknowledge, however, the conundrum that even if academic researchers might want people with learning disabilities to challenge rather replicate their research assumptions, the people with learning disabilities might want training in research that will help their confidence and employability.

There is an alternative to training self‐advocate researchers to use the skills and academic processes that academic researchers use, and that is to develop new methods together that work to form a good fit to the research needs, and that require collaborative thinking rather than transfer of skills and knowledge from expert to novice. This is described by Bigby *et al*. (2014):

Research methods were adapted to build on the strengths and skills of group members, take account of their limitations, and provide the type of support needed to work effectively together. Adapted methods evolved through a continuous process of reflection and adjustment.

They go on to describe how ‘by fleshing out what interviewees said, self‐advocates [researchers in this context] added their own knowledge and reflections to the data’ (p. 60) in a process of co‐construction. This is not what academic researchers are trained to do, and often not what they are in a position to do, but it is something that reflects the distinctive core of inclusive research. According to their account, the untrained but emergent processes enabled the ‘distinct contributions’ (p. 61) and distinctive perspectives of self‐advocate and academic researcher to be retained rather than blended or lost. This, we argue, is capacity building of a different kind, the value of which needs better recognition.

## Conclusion

The push and pull of wanting training for people with learning disabilities and wanting to retain their valuable insider or lay person perspective seeped through the seminars. Lou Townson and fellow self‐advocate researchers had previously got to the heart of the matter when they argued:

People who are not in the same boat as us don't understand what it is like to be us, they have not had our experiences. People with learning difficulties know that we have been through difficult times in our lives; we all have problems and have been mistreated. Because of this people want to talk to us. We know what they are talking about and understand them. (Townson *et al*. 2004, p. 73)

People with learning disabilities who become researchers do not lose their previous experiences, but they may come to see them differently. Hence, the common ground with other people with learning disabilities may shift a little. This is something we have only begun talking about. Not formally training people with learning disabilities as researchers will not result in them staying the same of course. It would be wrong of any academic researcher to advocate holding back researchers from outside the academy from their own transformation. Instead, we are advocating that this is further discussed as one of the difficult conversations that Walmsley & Johnson (2003) draw attention to the need for. It is our contention that more dialogue is called for about the model (or combination of models) of TCB that can best serve this particular push and pull emerging as inherent to inclusive research. It would be all too easy for discussions about necessary training for inclusive research to fall back on traditional roles regarding expertise, with knowledge and training in research skills flowing from the academic researchers to the researchers with learning disabilities. Partnerships, though, need to be reciprocal; training and capacity building, however it develops, should not undermine this.

## Notes

1 These contributions are in the public domain; they constitute a mix of published presentations and public discussion with an explicit view to extending the knowledge base. Hence, shared ideas were not data as such, and informed consent was not sought. Seminar contributors made a valuable contribution to our thinking, but the ideas in this paper are our own.

2 This and all the seminar presentations and summaries are available for download on the project blog: http://participat.blogspot.co.uk/
